# Oral switch antibiotic therapy in uncomplicated *Enterococcus faecalis* bloodstream infection

**DOI:** 10.1093/jacamr/dlaf004

**Published:** 2025-01-23

**Authors:** Sarah Al Mansi, Margaret Pokalsky, Katherine Turnley, Andrew Freeman, P Brandon Bookstaver, Joseph Kohn, Hana R Winders, Sarah Withers, Majdi N Al-Hasan

**Affiliations:** University of South Carolina School of Medicine, Columbia, SC 20203, USA; Department of Internal Medicine, Division of Infectious Diseases, Prisma Health Midlands, Columbia, SC, USA; University of South Carolina School of Medicine, Columbia, SC 20203, USA; University of South Carolina School of Medicine, Columbia, SC 20203, USA; University of South Carolina School of Medicine, Columbia, SC 20203, USA; Department of Clinical Pharmacy and Outcomes Science, University of South Carolina College of Pharmacy, Columbia, SC, USA; Department of Pharmacy, Prisma Health Midlands, Columbia, SC, USA; Department of Pharmacy, Prisma Health Midlands, Columbia, SC, USA; Department of Pharmacy, Prisma Health Midlands, Columbia, SC, USA; Department of Pharmacy, Prisma Health Upstate, Greenville, SC, USA; University of South Carolina School of Medicine, Columbia, SC 20203, USA; Department of Internal Medicine, Division of Infectious Diseases, Prisma Health Midlands, Columbia, SC, USA

## Abstract

**Background:**

The role of oral switch antibiotic therapy in uncomplicated *Enterococcus faecalis* bloodstream infection (BSI) remains unclear. This retrospective cohort study examines the effectiveness of oral switch compared with standard intravenous antibiotic therapy in uncomplicated *E. faecalis* BSI.

**Methods:**

Adults with first episodes of uncomplicated monomicrobial *E. faecalis* BSI were admitted to 10 Prisma Health hospitals in South Carolina from January 2021 to June 2023 were included. Deaths within 7 days were excluded to mitigate immortal time bias. Multivariate Cox proportional hazards regression examined the risk of treatment failure (all-cause mortality or recurrence) within 90 days after adjustment for the propensity of transitioning to oral switch therapy.

**Results:**

Of 476 screened patients, 131 with uncomplicated *E. faecalis* BSI were included in the analysis. The median age was 70 years, 84 (64%) were men, and 46 (35%) had a urinary source of infection. Eighty-seven patients (66%) received standard intravenous therapy and 44 (34%) were transitioned to oral switch therapy. Aminopenicillins were the most commonly used antibiotics for oral switch therapy (33/44; 75%). There was no increased risk of treatment failure with oral switch therapy compared with standard intravenous therapy (hazard ratio 0.77, 95% CIs 0.23–2.57, *P* = 0.67). Hospital length of stay (HLOS) was 7 and 11 days in the oral switch and standard intravenous groups, respectively (*P* < 0.001).

**Conclusions:**

Transitioning patients with uncomplicated *E. faecalis* BSI from intravenous to oral switch antibiotic therapy appears to be a promising strategy with shorter HLOS and no significant increase in the risk of treatment failure.

## Introduction

The burden of bloodstream infections (BSI) in North America and Europe is estimated at nearly 2 million episodes and 250,000 deaths annually.^1^  *Enterococcus* species accounts for 9% of BSI and ranks third among hospital-acquired BSI.^2,3^ Inappropriate antimicrobial therapy is associated with increased mortality in *Enterococcus* species BSI, making it essential to consider the full clinical context, including complications and comorbidities, when deciding on a treatment plan.^3^ The effectiveness of transitioning patients from intravenous to oral antibiotic therapy has been demonstrated in gram-negative BSI.^4–7^ More recent studies demonstrated that there was no statistical difference in treatment failure rates between oral switch and intravenous antibiotic treatment in BSI due to *Streptococcus* species.^8–11^ There is also emerging literature on the role of oral switch therapy in low-risk patients with uncomplicated *Staphylococcus aureus* BSI.^12,13^ Consistently, oral switch antibiotic treatment was associated with a shorter hospital length of stay (HLOS) in patients with BSI.^8–12^ The early transition from intravenous to oral antibiotics may also reduce costs associated with outpatient parenteral antibiotic therapy and may spare many patients from potential peripherally inserted central venous catheter complications.^10,14^ The role of oral switch antibiotic therapy in uncomplicated *Enterococcus faecalis* BSI remains unclear. This retrospective multi-hospital cohort study examines the effectiveness of oral switch antibiotics compared with standard intravenous therapy in adult patients with uncomplicated *E. faecalis* BSI.

## Materials and methods

### Settings

Adults with uncomplicated *E. faecalis* BSI admitted to 10 Prisma Health hospitals from January 2021 to June 2023 were evaluated. The Prisma health-system consists of community and community-teaching hospitals serving both rural and urban communities across 21 counties in South Carolina. The 10 hospitals combine for over 2800 licensed beds. This study was approved by the Prisma Health Institutional Review Board.

### Definitions

The standard intravenous therapy was defined as receiving only intravenous antibiotics for the entirety of the treatment course with a minimum of 7 days of therapy. Oral switch therapy was defined as the transition from intravenous to oral antibiotics within 3–9 days from index *E. faecalis* BSI for a minimum of 4 days of oral antibiotic therapy.^12^ A recurrent BSI was defined as a BSI within 90 days of the index BSI that was due to the same genus and species of bacteria. A complicated BSI was defined as a persistent BSI after 72 h or infection at metastatic sites such as infective endocarditis, septic arthritis and osteomyelitis. Polymicrobial BSI was defined as the growth of more than one species of bacteria in a blood culture excluding potential skin contaminants such as coagulase-negative staphylococci. Immune-compromised hosts were defined as those with neutropenia (neutrophil count <500 cells/mL), recent use of steroids or chemotherapy in the last 30 days, HIV infection with a CD4 count <200 cells/μL, or transplant recipients. The primary outcome was treatment failure, defined as all-cause mortality or *E. faecalis* BSI recurrence within 90 days of the index BSI. We anticipated that patients with uncomplicated *E. faecalis* BSI may be transitioned from intravenous to oral antibiotics around Day 4 of therapy.^10,12^ Accordingly, the early clinical failure criteria (ECFC) were used to determine acute severity of illness at the likely time of that intravenous to oral transition.^15^ The ECFC are determined within 72–96 h of index BSI and have been validated to predict mortality in patients with *Enterococcus* species BSI.^16^ The ECFC includes systolic blood pressure <100 mmHg or vasopressor use, heart rate >100 beats per minute, respiratory rate ≥22 breaths per minute or mechanical ventilation, altered mental status and peripheral white blood cell count >12 000/mm^3^.^15,16^

### Case ascertainment

This multi-hospital retrospective cohort study evaluated adult patients (age ≥18 years) hospitalized with uncomplicated *E. faecalis* BSI. Exclusion criteria were polymicrobial, complicated and recurrent episodes of BSI. Patients who received <7 days of antibiotic therapy were excluded. Patients who died within 7 days of the index BSI were also excluded to reduce the impact of immortal time bias.

### Statistical analysis

Descriptive statistics were used to summarize the data: medians and interquartile ranges for continuous variables, counts and percentages for categorical variables. The primary aim was to examine the treatment failure in patients who received standard intravenous and oral switch antibiotic therapy. HLOS was examined as a secondary outcome.

Since treatment allocation was not randomized in this observational cohort study, a propensity score analysis was performed to adjust for potential variables that likely influenced the decision to use oral switch antibiotic therapy. Multivariate logistic regression analysis was used to identify variables that were independently associated with the receipt of oral switch therapy. Variables associated with oral switch therapy in univariate analysis (*P* < 0.05) were included in the multivariate logistic regression model. Odds ratios (OR) with 95% CI were reported to describe the strength of association between each variable and the propensity of receiving oral switch antibiotic therapy.

The primary outcome of treatment failure was evaluated using Kaplan-Meier survival analysis and Cox proportional hazards regression. Patients were followed for 90 days after the index BSI or until death. This method allowed for censoring patients who were lost to follow-up within 90 days on the day of their last healthcare encounter. After adjustment for the propensity of receiving oral switch antibiotic therapy, the risk of treatment failure was assessed using a multivariate Cox proportional hazards regression. A univariate Cox proportional hazards model was used to examine risk factors for treatment failure. The multivariate Cox proportional hazards model included the variable of interest (oral switch versus standard intravenous therapy), the propensity to receive oral switch therapy, and variables associated with treatment failure in the univariate Cox model (*P* < 0.10) using backward selection. Hazards ratios (HR) and 95% CI were reported to describe the strength of association between each variable and the risk of treatment failure. Wilcoxon rank sum test compared HLOS between the two groups.

JMP Pro (version 17.0, SAS Institute Inc., Cary, NC, USA) was used for the statistical analysis. The level of significance for statistical testing was defined as *P* < 0.05 (two-sided) unless otherwise specified.

## Results

### Clinical characteristics

**Figure 1.**
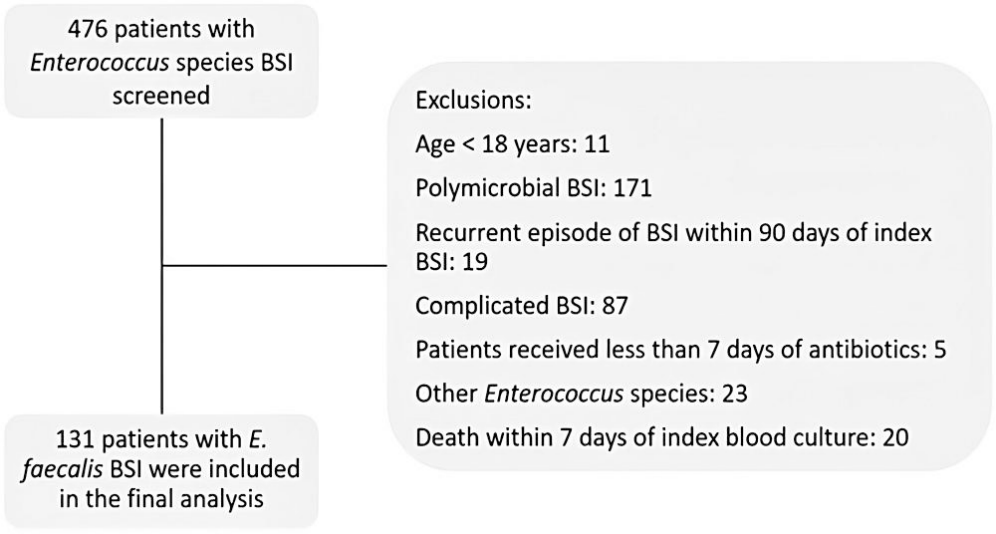
Study population and exclusion criteria.

Of 476 screened patients, 131 with uncomplicated *E. faecalis* BSI were included in the analysis (Figure 1). Overall, the median age was 70 years, and 84 (64%) were men. The urinary tract was the most common source of infection (46; 35%), followed by intra-abdominal (26; 20%), skin and soft tissue (12; 9%), central line-associated (11; 9%), other (12; 9%) and unknown source of infection (29; 22%). Complete source control was achieved in 15 of 26 patients (58%) with intra-abdominal infections within 96 h of *E. faecalis* BSI.

Eighty-seven patients (66%) received standard intravenous therapy and 44 (34%) were transitioned to oral switch antibiotic therapy. The baseline clinical characteristics of both groups are outlined in Table 1. In the univariate logistic regression model, cancer was associated with increased propensity of receiving oral switch antibiotic therapy. Diabetes mellitus, end-stage renal disease, the presence of an indwelling central venous catheter at the time of collection of index blood culture and infectious diseases consultation were associated with decreased propensity of receiving oral switch therapy. After adjustments in the multivariate logistic regression model, cancer (OR 3.61, 95% CI 1.29–10.12.61; *P* = 0.01), diabetes mellitus (OR 0.28, 95% CI 0.12–0.65; *P* = 0.004), the presence of a central venous catheter (OR 0.11, 95% CI 0.02–0.58; *P* = 0.008) and infectious diseases consultation (OR 0.26, 95% CI 0.11–0.63; *P* = 0.009) were independently associated with the propensity to receive oral switch antibiotic therapy and were included in the propensity adjustment model.

**Table 1.** Demographics and clinical characteristics of patients with *E. faecalis* BSI per treatment group

Variable	Oral switch therapy (*n* = 44)	Standard IV therapy (*n* = 87)	*P* value
Age, median (IQR)	71 (62–77)	70 (60–77)	0.52
Male sex, *n* (%)	28 (63%)	56 (64%)	0.93
Race, *n* (%)			0.06
White	33 (75%)	49 (56%)	
African American	9 (20%)	36 (41%)	
Other	2 (5%)	2 (2%)	
Diabetes mellitus, *n* (%)	15 (34%)	52 (59%)	0.006
End-stage renal disease, *n* (%)	2 (4%)	19 (21%)	0.01
Liver cirrhosis, *n* (%)	4 (9%)	7 (8%)	0.84
Cancer, *n* (%)	13 (29%)	10 (11%)	0.01
Nursing home residence, *n* (%)	5 (11%)	19 (21%)	0.14
Recent hospitalization within 90 days of index BSI, *n* (%)	28 (63%)	52 (59%)	0.67
Surgery within 30 days of index BSI, *n* (%)	13 (29%)	17 (19%)	0.21
Indwelling central venous catheter, *n* (%)	2 (4%)	22 (25%)	0.004
Indwelling urinary catheter, *n* (%)	9 (2%)	17 (19%)	0.90
Immune compromised host, *n* (%)	7 (15%)	9 (10%)	0.36
Urinary source of infection, *n* (%)	22 (50%)	24 (27%)	0.01
Echocardiogram performed	28 (63%)	67 (77%)	0.11
Infectious diseases consultation	23 (52%)	66 (76%)	0.006
ECFC, median (IQR)	1 (0–2)	1 (0–2)	0.14

IV, intravenous; IQR, interquartile range; BSI, bloodstream infection; ECFC, early clinical failure criteria.

### Antibiotic therapy

Antibiotic treatment durations were 15 and 14 days in the standard intravenous and oral switch therapy groups, respectively. Patients in the oral switch group received 6 days of intravenous antibiotics followed by 8 days of oral therapy. The oral antibiotics used were amoxicillin (25; 57%), amoxicillin/clavulanate (9; 21%), linezolid (5; 11%) and others (5; 11%). Various dosages and frequencies of administrations of oral amoxicillin were used: 500 mg three times a day (10), 500 mg twice a day (3), 500 mg four times a day (2), 875 mg twice a day (1) and 1000 mg three times a day (9). Aminopenicillin dosages were adjusted in six patients with a glomerular filtration rate < 30 mL/min/1.73 m^2^. All nine patients on oral amoxicillin/clavulanate received 875/125 mg dose twice a day. Oral linezolid was used at the standard 600 mg twice-a-day dose in all five patients.

### Clinical outcomes

**Figure 2.**
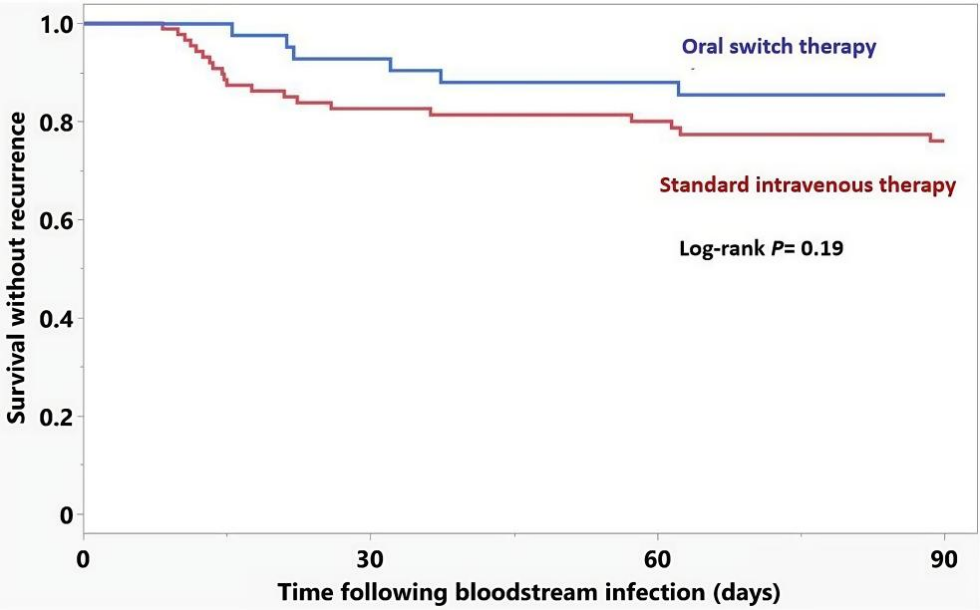
Kaplan-Meier curves for time to treatment failure by antibiotic treatment group.

The overall treatment failure within 90 days of uncomplicated *E. faecalis* BSI was 20.9% (26 deaths and no recurrences). Treatment failure was 14.5% in the oral switch and 24.0% in the standard intravenous antibiotic therapy groups (log-rank *P* = 0.19; Figure 2). The univariate Cox proportional hazards regression model demonstrated that a urinary source of BSI was associated with a lower risk of treatment failure, whereas ECFC were associated with a higher risk of treatment failure (Table 2). After adjustments for the propensity of receiving oral switch antibiotic therapy and other potential confounders in the multivariate Cox model, oral switch antibiotic therapy was not associated with an increased risk of treatment failure compared with standard intravenous therapy (HR 0.78, 95% CI 0.27–2.31; *P* = 0.66; Table 3). The independent risk factors for treatment failure included urinary source of infection (HR 0.27, 95% CI 0.08–0.93, *P *= 0.04) and ECFC (HR 1.64 per point, 95% CI 1.20–2.22, *P *= 0.001). HLOS was 7 days in the oral switch and 11 days in the standard intravenous antibiotic therapy group (*P* < 0.001).

**Table 2.** Univariate Cox proportional hazards regression model results for risk factors of treatment failure

Variable	HR	(95% CI)	*P* value
Age (per decade)	0.92	(0.71–1.22)	0.53
Male sex	1.60	(0.69–3.80)	0.29
White race	0.68	(0.31–1.47)	0.33
Diabetes mellitus	1.10	(0.51–2.37)	0.82
End-stage renal diseases	1.87	(0.79–4.46)	0.16
Liver cirrhosis	0.99	(0.23–4.18)	0.99
Nursing home residence	2.00	(0.84–4.78)	0.12
Recent hospitalization within 90 days	0.96	(0.43–2.12)	0.92
Surgery within 30 days	1.18	(0.49–2.80)	0.70
Indwelling central venous catheter	1.73	(0.73–4.13)	0.21
Indwelling urinary catheter	0.74	(0.26–2.16)	0.59
Cancer	1.15	(0.43–3.06)	0.78
Immune compromised host	0.90	(0.27–3.01)	0.87
Urinary source of infection	0.20	(0.06–0.68)	0.009
ECFC (per point)	1.79	(1.33–2.42)	<0.001
Oral switch antibiotic therapy (versus standard intravenous therapy)	0.55	(0.22–1.37)	0.20

HR, hazards ratio; CI, confidence intervals; ECFC, early clinical failure criteria.

**Table 3.** Independent risk factors for treatment failure in multivariate Cox model

Risk factor	HR	(95% CI)	*P* value
Urinary source of infection	0.22	(0.06–0.77)	0.02
ECFC (per point)	1.61	(1.18–2.19)	0.003
Propensity of receiving oral switch antibiotic therapy	1.71	(0.34–9.59)	0.67
Oral switch antibiotic therapy (versus standard intravenous therapy)	0.77	(0.23–2.57)	0.67

HR, hazards ratio; CI, confidence intervals; ECFC, early clinical failure criteria.

## Discussion

### Accumulating evidence for the use of oral switch antibiotic therapy in BSI

Transitioning patients with uncomplicated *E. faecalis* BSI from intravenous to oral switch antibiotic therapy was not associated with an increased risk of treatment failure in this multi-hospital cohort study. Moreover, oral switch therapy had the advantage of a shorter HLOS compared with standard intravenous antibiotic therapy. Transitioning from intravenous to oral antibiotic therapy has become the standard of care in the management of gram-negative BSI.^6^ However, intravenous antibiotic therapy has remained the traditional method of treatment for gram-positive BSI. Multiple observational cohort studies have demonstrated favourable prognosis with similar clinical outcomes when comparing intravenous and oral-stepdown therapy in uncomplicated streptococcal BSI.^8–11^ One observational cohort and a randomized controlled trial showed non-inferiority of early oral antibiotic switch therapy in low-risk *S. aureus* compared with standard intravenous therapy.^12,13^ The utility of this strategy remains unclear in *E. faecalis* BSI.

There is scarce evidence supporting this approach in *E. faecalis* BSI. To date, no randomized controlled trials have been published to demonstrate treatment outcomes of oral switch antibiotic therapy versus standard intravenous therapy in uncomplicated *E. faecalis* BSI. To our knowledge, this is the second observational cohort study to examine the effectiveness of oral switch antibiotic therapy in *E. faecalis* BSI. In the first study on this topic, Loudermilk *et al*. examined 30-day treatment outcomes in patients with uncomplicated *E. faecalis* BSI, comparing intravenous to oral switch antibiotic therapy in a multicenter, retrospective and matched cohort.^17^ There was no significant difference between the two groups in the composite primary outcome of 30-day mortality, microbiologic failure, clinical failure or 30-day readmission (14.5% versus 21.8%; OR 0.53, 95% CI 0.23–1.25). Patients in the oral switch group were transitioned to oral antibiotics after 6 days of intravenous therapy. HLOS was significantly shorter in the oral switch group compared with the traditional intravenous therapy group (6 versus 14 days; *P* < 0.001.)^17^ These findings were consistent with the results of the current investigation. Oral antibiotic selection was also comparable in the two cohorts. Two-thirds of patients in the oral switch group were transitioned to oral aminopenicillins (amoxicillin and amoxicillin/clavulanate)^17^ as compared with three-quarters of patients in the current study.

### Potential impact on clinical practice

Based on the results of the current and the previous cohort,^17^ it appears that transitioning patients with uncomplicated *E. faecalis* BSI from intravenous to oral antibiotic therapy may be a feasible and safe strategy in selected patients. Obtaining an echocardiogram and other appropriate workup is essential to rule out complications prior to transitioning to oral switch therapy.^18^ A favourable early response to initial intravenous antibiotic therapy, as determined by the ECFC, is recommended prior to the transition to oral therapy.^15,16^ It has been demonstrated in the current and a previous study that patients with *E. faecalis* BSI and ECFC ≤2 after 72–96 h of antibiotic therapy have a lower risk of mortality and treatment failure.^16^ Similarly, patients with a urinary source of *E. faecalis* BSI have a lower risk of treatment failure and may be more appropriate for transitioning from intravenous to oral antibiotics than those with intra-abdominal or unknown source of infection.

Regarding the most appropriate antibiotics for oral switch transition, aminopenicillins were by far the most commonly used class of antibiotics in both the current and previous study.^17^ Quinn *et al*. demonstrated no significant difference in treatment failure rates using oral switch antibiotic regimens with varying oral bioavailability in a cohort of non-staphylococcal gram-positive BSI, including 10 patients with *Enterococcus* species.^19^ From the antimicrobial stewardship standpoint, oral amoxicillin is preferred over amoxicillin/clavulanate in aminopenicillin-susceptible *E. faecalis* BSI to avoid unnecessarily broader coverage and gastrointestinal adverse effects associated with clavulanate. A previous pharmacokinetic/pharmacodynamic model demonstrated that 1000 mg of amoxicillin every 6 h is ideal for achieving target concentrations in the serum and interstitial cells of the kidneys, which was the dose of amoxicillin used in the POET trial.^20,21^ Neither the current nor the previous cohort had adequate power to examine the effectiveness of the various dosages of amoxicillin used.^17^ Future larger studies are likely needed to determine the most appropriate dose of aminopenicillins in this setting. Amoxicillin/clavulanate may be appropriate in patients with *E. faecalis* BSI originating from an intra-abdominal source, following adequate source control, as it offers coverage for anaerobic bacteria due to the polymicrobial nature of these infections. Oral linezolid may be an option for patients with amoxicillin allergy or those who may have difficulty adhering to a triple or quadruple dose-per-day regimen. A randomized controlled trial would be beneficial before the widespread implementation of oral switch antibiotic therapy in uncomplicated *E. faecalis* BSI.^22^

Moreover, the total antibiotic treatment duration in *E. faecalis* BSI remains undetermined. A recent randomized controlled trial demonstrated non-inferiority of 7 days of antibiotic therapy for BSI compared with 14 days.^23^ However, the overall results were mostly driven by gram-negative BSI and non-inferiority of 7 days of therapy was not achieved in gram-positive BSI in the subgroup analysis of the trial.^23^ It is possible that the investigation was not adequately powered to examine the outcomes separately in gram-positive BSI. However, it is too preliminary at this point to suggest 7 days of total antibiotic therapy for *E. faecalis* BSI based on the results of the BALANCE trial. An ongoing clinical trial is currently examining the appropriate treatment duration in *Enterococcus* species BSI.^22^

### Strengths and limitations

The use of propensity score analysis added strength to the study as it allowed adjustments for the variables that influenced the transition from intravenous to oral switch antibiotic therapy without overcrowding the final Cox model. Utilization of ECFC to determine acute severity of illness and predicted prognosis at the time of potential transition from intravenous to oral antibiotic therapy represents a unique approach. Most previous studies examining the transition from intravenous to oral antibiotics for BSI have only accounted for acute severity of illness at the onset of BSI, which may vary by the time patients are transitioned to oral therapy.^6^ The exclusion of patients who died within 7 days of BSI helped mitigate the risk of immortal time bias. The use of 90-day treatment failure as the primary outcome of the study is consistent with the recommendations of a consensus panel of experts on BSI.^24^

There were several limitations to the current study. First, the observational cohort design lacked randomization of treatment allocations. The decision to transition to oral switch antibiotic therapy was made at the primary healthcare provider’s discretion. The effects of unmeasured and unknown confounders might persist despite adjustments in the multivariate Cox model and the use of propensity score analysis. The variables were passively collected from the electronic medical records. While the data on dosing and duration of antimicrobial therapy were available, adherence to these regimens could not be assessed. Additionally, multiple dosages of oral amoxicillin were used. Examination of the most appropriate dose of oral amoxicillin lacks adequate power. Lastly, the study included 10 hospitals across South Carolina within one healthcare system. This may influence generalizability to other geographic and clinical settings.

## Conclusions

The current study results provide additional evidence that transitioning from intravenous to oral switch antibiotic therapy may be a promising strategy in selected patients with uncomplicated *E. faecalis* BSI. Shorter HLOS in association with oral switch therapy compared with standard intravenous antibiotics without a significant increase in the risk of treatment failure makes this strategy more appealing to patients, healthcare providers, and healthcare systems. Confirmation of these results in future larger clinical studies, including randomized controlled trials, would improve generalizability and widespread implementation of this treatment strategy.
